# Bioanalytical automatlon: history and future plans

**DOI:** 10.1155/S1463924694000118

**Published:** 1994

**Authors:** Raymond H. Farmen

**Affiliations:** Bioanalylical & Radiochemical R & D Bristol-Myers Squibb 675 College Road East Princeton New Jersey 08543-4500 USA

## Abstract

Bioanalysis is determining the concentration of drugs and metabolites in biological fluids (i.e. plasma and urine). During the past 15 years tremendous advances in bioanalysis, for example HPLC, auto injectors, data collection systems and robotics has enabled the productivity of the bioanalyst to increase but it still requires considerable manual intervention. This paper describes the rationale, the justification and the plans Bristol-Myers Squibb has to completely automate the entire bioanalytical process.


Journal of Automatic Chemistry, Vol. 16, No. 4 (July-August 1994), pp. 121-123

### Bioanalytical automation: history and future plans

**Raymond H. Farmen**
*Bioanalytical & Radiochemical R & D, Bristol-Myers Squibb, 675 College Road East, Princeton, New Jersey 08543-4500, USA*

*Bioanalysis is determining the concentration of drugs and metabolites in biological fluids (i.e. plasma and urine). During the past 15 years tremendous advances in bioanalysis, for example HPLC, auto injectors, data collection systems and robotics has enabled the productivity of the bioanalyst to increase but it still requires considerable manual intervention. This paper describes the rationale, the justification and the plans Bristol-Myers Squibb has to completely automate the entire bioanalytical process.*

Fifteen years ago bioanalysis was extremely tedious. Quantitating drugs in plasma and urine was accomplished by either RIA or GC. Although RIA was non-specific, and raising appropriate antibodies bordered on black magic, this was often preferred over the labour-intensive GC. GC required tedious chemical derivatization with manual injection, followed by manual chromatographic integration. When HPLC was introduced in the 1970s, it proved to be a fantastic technological advance in bioanalysis because it offered specificity without the need for chemical derivatization. Unfortunately, HPLC still required manual injection and manual chromatographic integration. When autoinjectors and chromatographic integrators became available they quickly increased the productivity of both GC and HPLC. These advances only addressed the obvious needs of the chromatographic portion of sample bioanalysis. However, sample extraction and preparation was still performed manually. The introduction of fast LC systems with air bubbles as separators was an attempt to automate the sample extraction portion of bioanalyis. Unfortunately, this technology did not prove to be robust. Zymark's robotic stations were successful at automating the sample extraction steps.

Many people consider their bioanalytical laboratories automated if they have Zymark robots, automatic injectors and data reduction systems. As shown in figure 1, there are many steps involved in bioanalysis and the commercially available equipment will only address the sample analysis step. Clearly, considerably more can be automated in this bioanalytical process. In fact, the entire system should be able to be automated if it were economically beneficial.

During 1992 Bristol-Myers Squibb generated about 200 000 bioanalytical samples that needed to be analysed. Of these samples about two-thirds of them were analysed in-house and the remainder were sent to contract laboratories. Without the use of robotics, the typical in-house analyst can prepare a bioanalytical run every other day. The average bioanalytical run contains about 90 injections, 20 of which are standards and quality control samples. Therefore, the typical analyst can process about 175 unknown samples per week. During 1992 about 63 000 samples were sent to contract bioanalytical laboratories for bioanalysis for two reasons. First, Bristol-Myers Squibb has inadequate facilities for handling samples known to be HIV+. Therefore, any known HIV+ sample is sent out for bioanalysis. Second, with the current level of automation within the bioanalytical department it was impossible to handle the tremendous quantity of samples. It should be stressed that sending samples to contract laboratories for bioanalysis only saves about two thirds of the resources that would be used for in-house bioanalysis. The analyst in Bristol-Myers Squibb is still responsible for verifying the bioanalytical results and for getting the data in a format for subsequent pharmacokinetic analysis. It costs on average about $38 per sample for outside contract bioanalysis. However, the *real* cost of sending samples to contract laboratories for bioanalysis is $60 per sample, taking into account both the contract and in-house costs. During 1992, Bristol-Myers Squibb spent $2.4 M dollars on bioanalytical contracts.

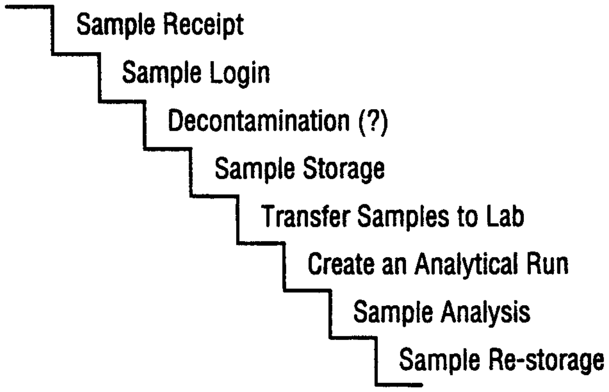

*Figure 1. Sample handling steps.*

### Total automation

If the entire bioanalytical process were to be automated at Bristol-Myers Squibb, the goals would be:

(1) Increase productivity—go from 175 to 1500 samples/week/analyst.

(2) Increase capacity—plan to process 400 000 samples/year.

This paper was presented at the 1993 ISLAR, organized by the Zymark Corporation.

© Zymark Corporation 1994

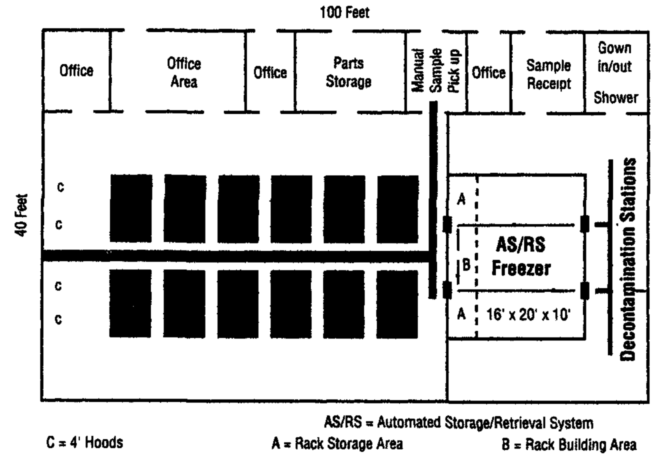

*Figure 2. Bioanalytical laboratory layout.*

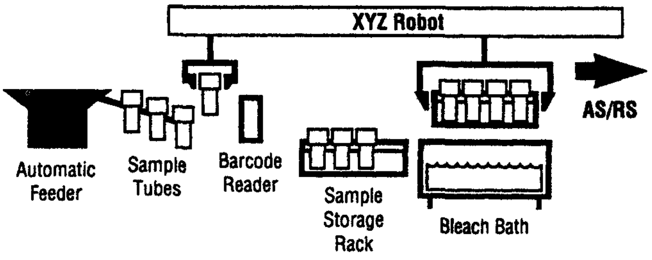

*Figure 3. Sample login and decontamination station.*

(3) Reduce reliance on contract laboratories and save millions of dollars each year.

(4) Improve analyst safety from blood-born diseases and be able to bring HIV+ samples in-house for bioanalysis.

(5) Maximize the time that expensive equipment is utilized by continuously feeding it samples.

(6) Accelerate drug development by processing samples at a faster rate.

(7) Improve our sample tracking capabilities that are often done manually.

(8) Improve worker morale.

A 40 ft × 100 ft automation bioanalytical laboratory that addresses all of the sample processing steps described in figure 1 is shown in figure 2. Sample flow in this laboratory moves from right to left. Sample receipt and its subsequent decontamination occur in the room on the right.

A schematic for the automated sample login and decontamination station is shown in figure 3. Following decontamination the samples are sent into a large freezer for storage at −20°C. This freezer has been named the 'AS/RS'—the automatic storage and retrieval system. The freezer has two linear tracks on which robots move to place on and pick off 50 tube trays from storage racks. This process can be viewed very much like an automated warehouse, only it is smaller and colder. During sample bioanalysis, selected samples are removed from their trays in the freezer and placed into 20 tube trays for transport to the bioanalysis stations. The trays are transported to

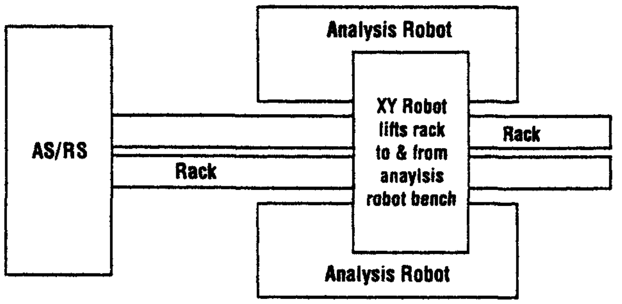

*Figure 4. Sample transport conveyor system.*

the bioanalysis stations by simple conveyor belts. The trays are removed from the conveyor belts by a robot and placed on the bioanalysis station. After an aliquot has been taken from each sample in the tray, the tray is then placed back on the conveyor belt for transport back to the AS/RS for storage. A schematic of the transport system shown in figure 4. The final step in the automation process (sample bioanalysis) has already been fully automated. The sample bioanalysis process consists of extraction, chromatographic separation, measurement and quantitation.

Managing this automation laboratory will require considerably different skills than are currently envisioned in a research environment. For example, the goal of this laboratory is to run 24 hours a day, seven days a week. Managing that work will require production management as well as scientific management training. It is anticipated that the entire automation laboratory with 12 bioanalysis stations will require a staff of six bioanalytical scientists, two computer scientists, one decontamination technician and one manager. That is two bioanalysis stations per scientist. Handling the current sample workload would be simple in this laboratory. For example, if 11 of the 12 bioanalysis stations process one sample every 15 minutes, 24 hours a day, five days per week and 40 weeks per year, the result would be well over 200 000 samples being analysed. Sample capacity could easily be increased by either increasing sample throughput per bioanalysis station, and/or by working more days/week, and/or by adding more bioanalysis stations. Clearly, increasing sample throughput would be the preferred choice.

The entire cost estimate of this automation laboratory ranges from \$4 500 000 to \$7 000 000. The hardware alone will cost about \$2 500 000 with software/engineering costing between \$2 000 000 to \$4 500 000. The large range in software expense is dependent upon what internal resources can be devoted to this project. The question that is often asked is whether this automation laboratory will be economically justifiable? Figures 5 and 6 address this question. Figure 5 compares the per sample bioanalytical cost between automated sample bioanalysis versus manual sample bioanalysis. As the number of samples increases, the cost of performing automated bioanalysis decreases to about \$38 per sample. The figure of \$38 per sample amount represents the high side of total sample bioanalysis because the scientist performing the bioanalysis was still responsible for manually logging in the samples, for tracking the samples, for transporting the

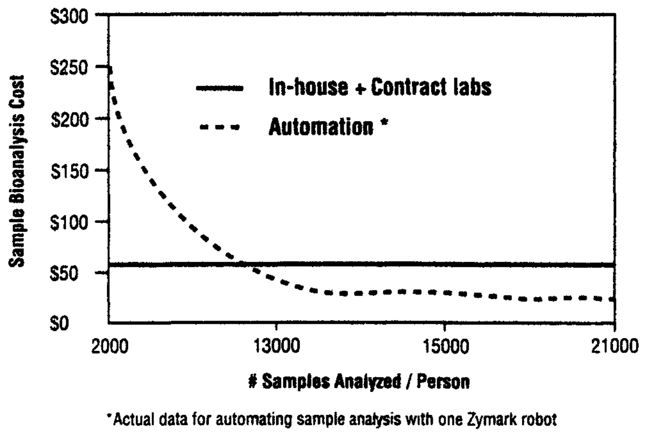

*Figure 5. Bioanalytical cost comparison.*

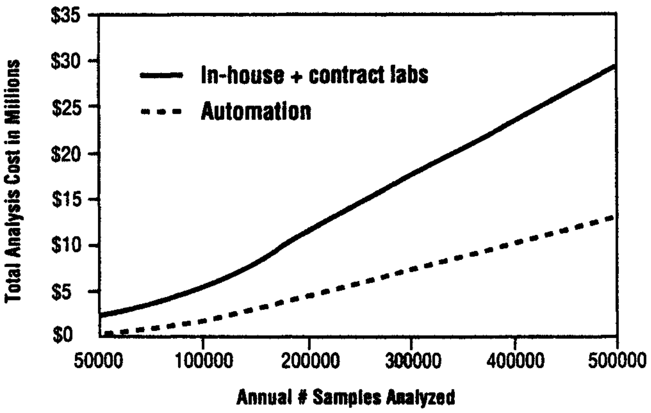

*Figure 6. Bioanalytical cost comparison.*

samples to the laboratory and for setting up the analytical run. In figure 6 the total sample bioanalysis cost in millions of dollars, versus the annual number of samples analysed in-house, is plotted comparing the in-hour/contract cost of $60 per sample against the automation sample bioanalysis cost of $38 per sample. These cost comparisons were used to perform a return on investment analysis. Under the worst case scenario that the total cost of the automation laboratory was 35% more than $7 000 000, that the efficiency of the automation lab was 35% less than projected (efficiency was one sample processed every 15 minutes per sample bioanalysis station) and that the sample capacity stayed the same, this system would pay for itself in less than three years. Creating this bioanalytical automation laboratory produced several new and excisting challenges. First, this laboratory will force the company to redefine a bioanalytical experiment. For example, today's bioanalytical experiment consists of 11–12 standards, 6–8 quality control (QC) samples and 60–100 unknown study samples. In the continuous feed automation laboratory the potential exists for a bioanalytical experiment to contain 500–1000 samples. In an experiment of this size how many standards and QCs are appropriate? Also, how should the standards and QCs be placed in the run? Second, chromatographic software will need to be developed that can diagnose problems with the experiment and can then terminate that experiment. Third, LCMS machines will be interfaced with this system. This equipment has the ability to accept a new sample every four minutes. With the continuous-feed automation laboratory, the rate limiting step is the sample extraction process. Manufacturers will need to develop technology that greatly increases the speed of that bioanalytical step.

### Conclusion

In conclusion, a vision for automating bioanalysis has been presented. This vision moves away from the historical perspective that the only steps in bioanalysis that need to be automated are the sample extraction step, the sample separation and measurement step and the sample quantitation step. This vision offers the potential for increased productivity, increased capacity and increased worker safety. This vision was turned into a plan; albeit an expensive plan but with the potential for big monetary savings. This plan in a relatively low-risk plan, because similar systems have been successfully implemented such as warehousing and clinical chemistry operations.

